# Physical Therapy Interventions of an Electrical Burn Injury-Afflicted Patient: A Case Report

**DOI:** 10.7759/cureus.31176

**Published:** 2022-11-06

**Authors:** Sana A Shaikh, Priyanka A Telang, Sakshi Arora

**Affiliations:** 1 Physiotherapy, Ravi Nair Physiotherapy College, Datta Meghe Institute of Medical Sciences, Wardha, IND

**Keywords:** physiotherapy in burns, rehabilitation, community health, electrical injury, pain management, burns

## Abstract

Electrical injuries are uncommon but not completely rare. It is most prevalent in the male population, although females are also affected in the workplace or household-related activities. These injuries usually occur in situations where proper precautions are not taken by the individual and also appropriate safety drills and education for personnel are not carried out. Electrical burns affecting children are very rare, but when they do occur, it is usually due to accidental contact with exposed electrical sources. In this patient, there were severe levels of secondary complications following the burn injury. The patient developed blood infections and also was hampered in doing a variety of activities of daily living. The patient was diagnosed with 45%-50% body surface area (BSA) covered with burns, which suggests its severe nature. Treatment focuses on preventing wound infection, managing the excruciating amount of pain, preventing complications of immobility, promoting mobility as much as the patient can, and also educating the patient and the family members.

## Introduction

Burn injuries are a form of trauma to the body tissues that are less recognized as compared to other injuries. These injuries can occur to anyone regardless of age, gender, or location. These injuries can be contributed to several reasons such as friction, cold, heat, radiation, and chemical or electrical sources. Out of these, the majority of burns are caused due to hot liquids or close contact with a hot object. Although energy transfer results in tissue damage in all cases of burn injuries, distinct causes can be linked to various physiological and pathophysiological reactions [1].

The extent of the burn wound

To calculate the extent of burn a person has suffered, the rule of Wallace is used. The rule of Wallace, also commonly referred to as the rule of nines, is a frequently used outcome measure used by emergency and trauma care providers. It is used to assess the total body surface area (BSA), which is involved in burn injury. This rule of Wallace is specifically used in the assessment of second- and third-degree burns, which helps in determining the severity of the condition [2].

Pathophysiology of the burn

There are three major zones of burns, namely, the zone of coagulation, the zone of stasis, and the zone of hyperemia. The zone of coagulation is where the maximum damage has occurred [3]. The zone of stasis is characterized by the tissues that can be saved from progressing toward necrosis if timely managed by healthcare professionals [4]. The zone of hyperemia is where there is an adequate blood supply to the injured tissue and also there is an evident healing process [5].

Electrical burn injuries

Electrical injuries are uncommon, but when they occur, they result in devastating injuries. These are associated with a high rate of hospital admissions and deaths. These injuries constitute approximately 0.04%-5% of admissions in the burn departments in developed countries and up to 27% in developing countries [6]. Electrical injuries result in necrosis of the skin and the deeper tissue structures resulting in decreased function of the affected limb or even complete loss of function [7].

Critical burn rehabilitation plays a major role in the recovery process of the individual who has sustained a burn injury. The principal aim of rehabilitation in such patients is to maintain the range of motion, prevent contractures, improve strength, and also have adequate pain management [8].

## Case presentation

A 27-year-old male patient, on 11/06/2022, suffered from a severe electrocution burn injury while doing some official work. On the day of the injury, he was working with live wires on an electrical pole when suddenly the power transmission through the wires started, and he sustained a high-voltage electrical shock. He fell off the pole and went unconscious. Soon after the injury, the patient developed severe burns with excruciating pain. Upon hearing from the patient's relatives, the patient was taken to a local hospital because of an electrical burn. From there, he was referred to our hospital for further management. He was managed with IV fluids and later underwent skin graft transplant surgery. Postoperatively, physical therapy management was started to prevent primary and secondary complications.

Clinical findings

**Figure 1:**
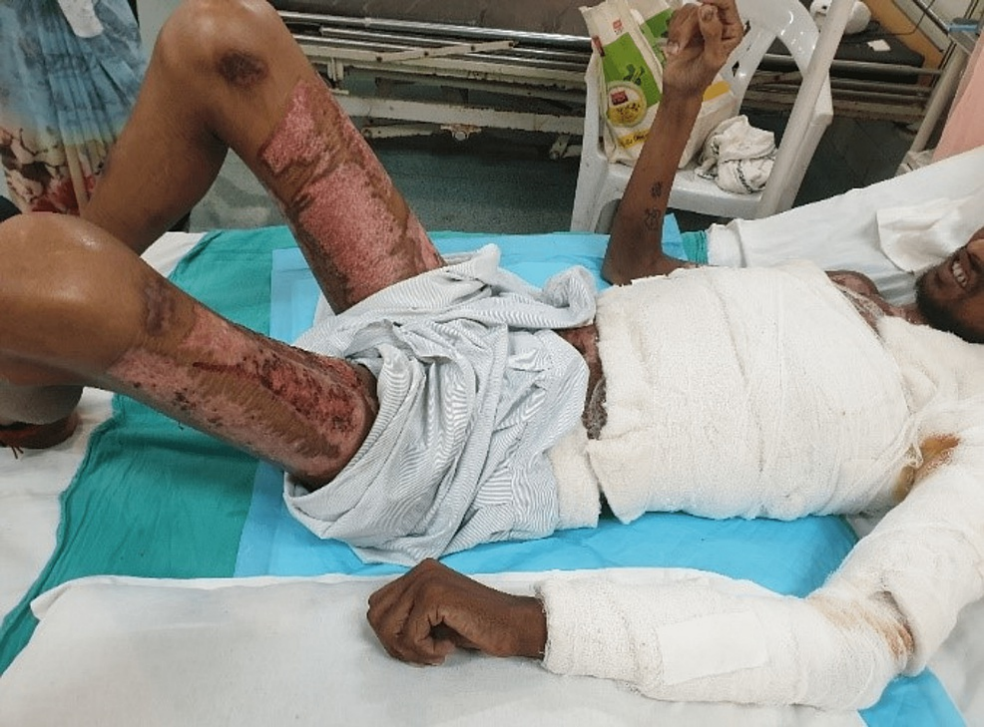
The patient is lying supine with bandages covering the burnt area. The right elbow is seen to be fixed in flexion contracture.

**Figure 2:**
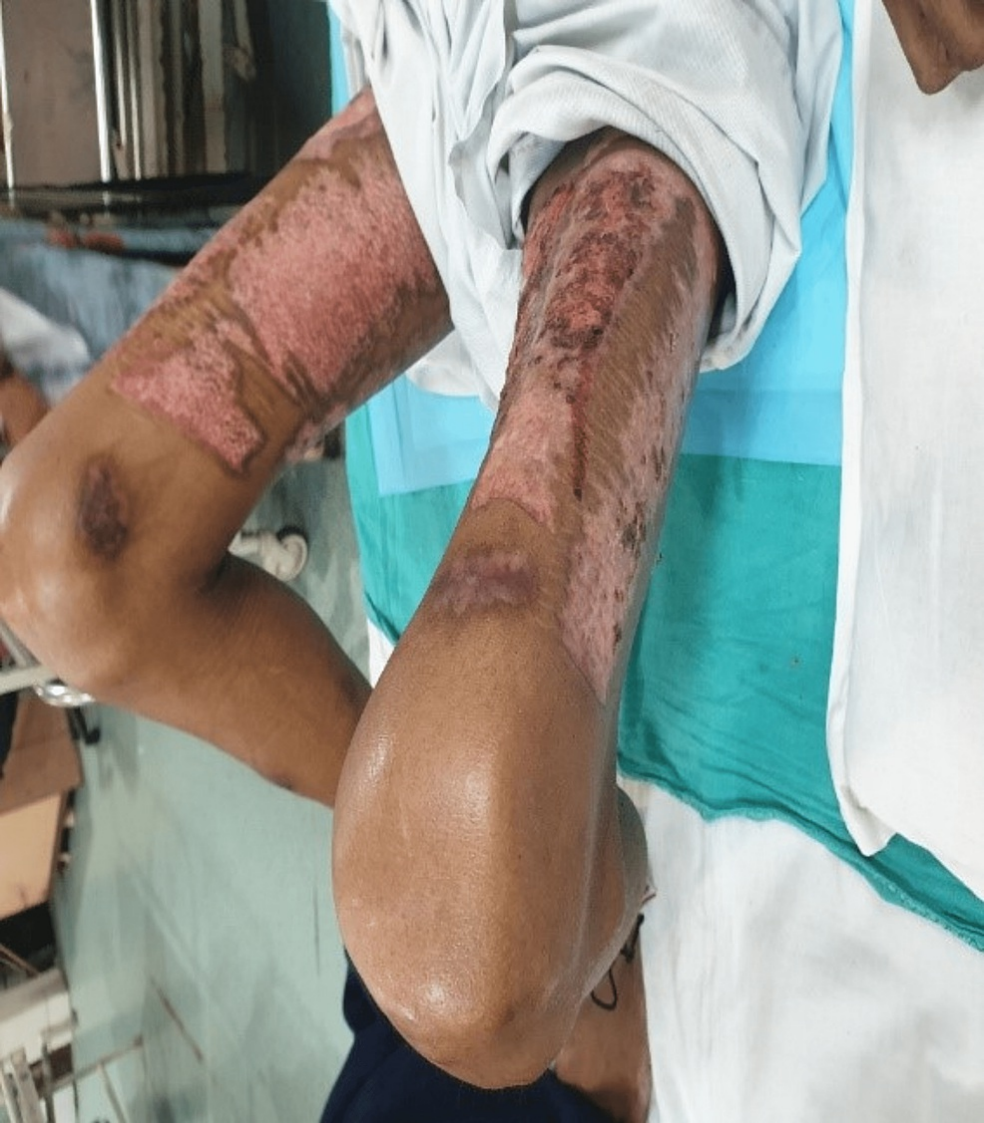
Scars from skin graft transplant surgery can be appreciated on both thighs (donor site).

Proper consent was taken from the patient and the relatives before performing the assessment. The patient was examined supine lying with the shoulder in a neutral position and right elbow in 90 degrees of flexion and hip and knee in flexion. On careful examination of the patient, the extent of the burn the patient has suffered throughout his body is calculated to be 45%-50% BSA. The patient had burns extending from the neck to just below the lower abdomen. The right upper extremity is involved from the shoulder up to the wrist joint. The left lumbar region and the lateral part of the back are also involved with third-degree burns. The level of pain was noted after consulting with the patient, which was 8/10 on the Numeric Pain Rating Scale (NPRS). The development of bilateral contractures was seen on the elbows. An early level of contracture was also seen in the neck region. Skin grafts were taken from both thighs for plastic surgery. Figure 1 shows the patient in bandages covering the majority of the burned part and flexion contracture of the elbow. Figure 2 shows skin graft scars on the thighs bilaterally.

Investigations

Investigations performed were complete blood count (CBC), electrolytes, blood cultures, urine analysis, and creatine phosphokinase. CBC showed a decrease in the number of red blood cells. Also, the blood cultures suggested a blood infection. Other investigations that were performed included a range of motion assessment, manual muscle testing, end-feel evaluation, and pain assessment.

Patient's concern

The patient complained of the inability to completely extend both elbows. He also complained of an inability to perform supination and pronation.

Assessment

Range of Motion

The patient is unable to perform a full-range extension of the elbow due to the presence of flexion contracture in the elbow. Also, the ability to pronate and supinate the forearm is hindered due to pain and scar tissue.

Manual Muscle Testing

The strength of the most affected muscles, which in this case are the upper limb muscles, is tested using the technique of manual muscle testing. It is done by applying resistance against the movement being performed. Table 1 shows the findings of the test.

**Table 1:** Manual muscle testing findings.

Joint assessed	Right	Left
Shoulder flexors	2/5	3/5
Elbow flexors	2/5	3/5
Wrist extensors	3/5	2/5

Diagnosis

After a thorough examination, the patient's diagnosis is concluded to be third-degree electrical burns.

Timeline

Table 2 shows the timeline of events from the day of injury up to the day when physiotherapy was started.

**Table 2:** Timeline of events.

Date	Event
11/06/2022	The patient suffered from an electrocution injury, developed severe burns, and was taken to a local hospital for management
18/06/2022	The patient was brought to our hospital for further management
20/06/2022	Skin graft plastic reconstruction surgery was performed
21/06/2022	Postoperative physiotherapy was started

Intervention

Physiotherapy management consists of two phases, namely, acute and chronic care. In acute care physiotherapy, the main aim is to prevent the complications of complete bed rest and also the management of pain. The acute care period can range anywhere from one day to four weeks postoperatively. In chronic care, the patient undergoes physiotherapy to prevent any kind of muscle wasting or weakness; it also focuses on improving the strength and mobility of the patient. This can last anywhere between two and four months after the initial injury or surgery.

Management

Week 1

**Figure 3:**
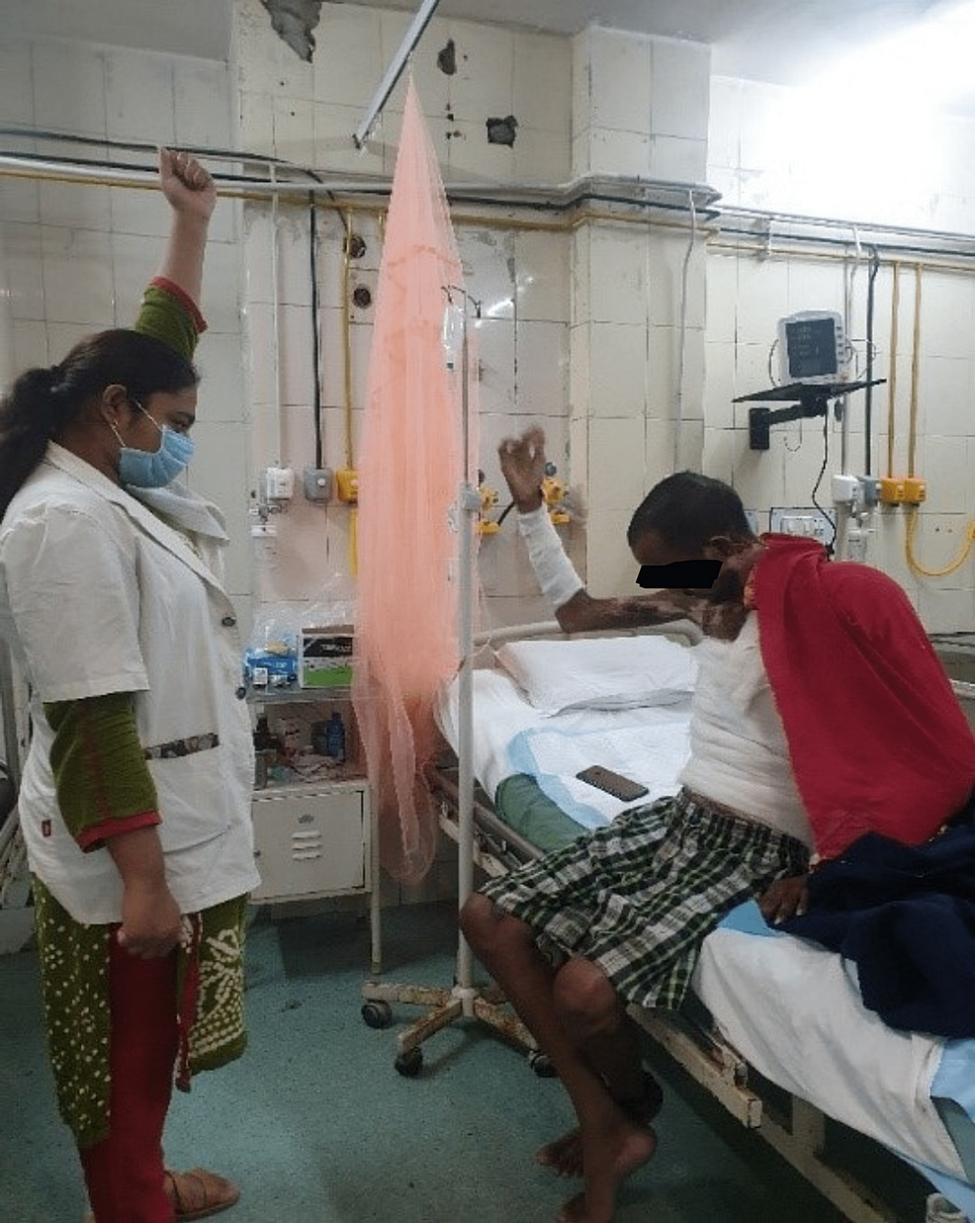
The patient is performing shoulder range of motion exercises.

**Figure 4:**
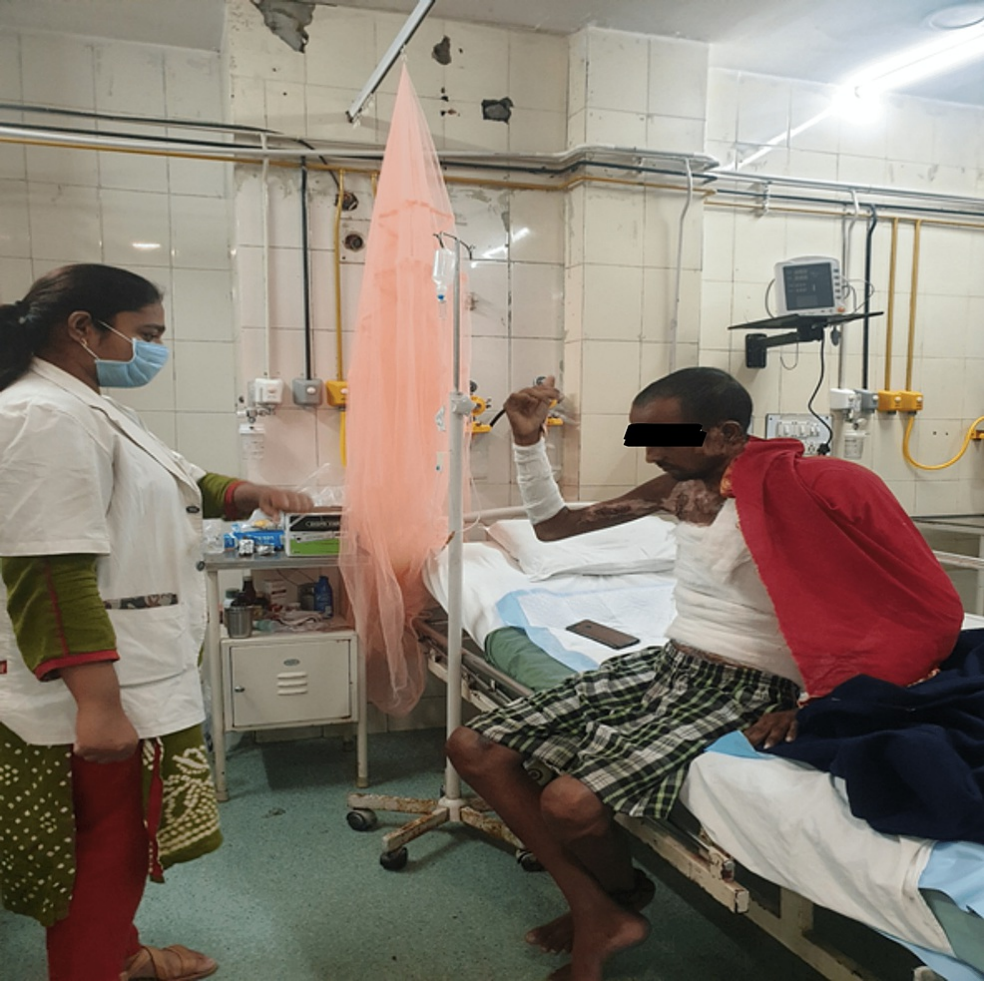
The patient is performing elbow range of motion exercises.

During the first week after surgery, the patient was referred for physiotherapy. The physiotherapy protocol was tailor-made by the therapist after keeping in mind the clinical findings and also the current status of the patient. The patient's protocol for the first week includes a light range of motion exercises such as ankle-toe movements, heel slides, and active shoulder, elbow, and wrist movements up to the pain-free range. Pursed lip breathing was done by the patient along with thoracic expansion exercises to maintain thoracic mobility and prevent the accumulation of chest secretions. These exercises were done by the patient every day for one week. Figures 3-4 show the patient performing active shoulder and elbow range of motion exercises.

Weeks 2-4

From the second week onward, passive range of motion exercises for the elbow joints that developed contractures were started. Passive flexion and extension up to the available range of motion were done by the therapist to prevent the progression of the contracture. Additionally, the sustained passive stretch was also performed to release existing contractures. Passive pronation and supination of the forearm are done to facilitate the said motion. Active finger movements were encouraged to be performed by the patient; these prevent edema and also prevent contractures. Static quadriceps and hamstrings are continued with the duration of hold increased to 10 seconds or as much as the patient can perform without inducing fatigue. Ultrasonic massage therapy can also be given at this stage to break adhesions.

Weeks 4-6

From week 4 onward, the patient is made to sit in high sitting position with his feet dangling on the bed. In this position, the patient is made to perform dynamic quadriceps. Shoulder protraction and retraction exercises were started to promote scapular mobility. Later during the fifth week, the patient is made to march at the spot, which is gradually progressed to hallway ambulation as much as the patient can. Transcutaneous electrical nerve stimulation (TENS) can be administered to the patient for pain relief. Strengthening exercises of the less affected limbs were started with weights. Active finger movements are continued along with pronation and supination exercises.

Home Exercise Program

The patient is asked to continue performing a range of motion exercises, i.e., active finger movements and active shoulder, elbow, and wrist range of motion. Self-stretches can also be taught to the patient, which can be easily performed by him daily. The patient is also educated on regularly monitoring vitals and should stop any kind of exercise if they are unstable. The patient is informed about the necessity to visit for physiotherapy follow-ups.

Follow-Up

During follow-ups, the patient can be given friction massage to prevent scar tissues and adhesions. Also, cyclic stretching can be given to break stubborn adhesions and also prevent contractures. Progressive resisted exercises can be started to further improve strength.

## Discussion

Electrical burns are very serious and can even prove to be fatal. These cases if neglected can lead to a variety of complications for the patient, which can ultimately result in high morbidity and mortality. These burns differ from thermal burns in the sense that they can affect the individual at different tissue and organ levels. An electrical burn may occur due to either direct contact with a current source or the reception of current by certain body tissues. Electrical burns rather have more systemic effects than just merely at the contact site [9]. In this patient, a variety of secondary complications arose due to negligence. The patient developed wound infections, which ultimately resulted in a blood infection.

Although he reports a history of alcohol addiction, as well as consumption of tobacco, the patient is otherwise fit and healthy. He underwent surgery for debridement and aesthetic purposes. Skin graft transplant is the most commonly performed surgery in both thermal and electrical third-degree burns. A full-thickness or split skin graft is irrigated from the donor site and placed at the recipient site. The main aim of the treatment of burn injuries is to prevent infections by covering or promoting the healing of the exposed part. The second important factor in the treatment of electrical burns is to promote early mobilization of whichever part of the body is affected [10]. Burn recovery is generally divided into two phases, namely, the early phase (or acute stage) and the rehabilitation phase (or late stage). The early phase starts from when the patient first reports to the hospital up to the time of wound debridement and closure, while the rehabilitation phase is from wound closure to scar maturation, which can take anywhere between eight and 16 months [11].

A proper physiotherapy protocol was made following a thorough assessment. The interventions included a variety of methods such as manual techniques, therapeutic exercises, active and passive exercises, and stretches, as well as certain electrotherapy modalities. These were followed by the patient every day for over a couple of weeks post injury, and other therapeutic interventions were continued for two to four months after the initial injury.

## Conclusions

Electrical burn injuries are one of the most complicated and gruesome injuries in the field of medicine and rehabilitation. These require constant patience and effort from the healthcare providers, patients, and patients' close ones. Throughout the course of the treatment, our patient was well motivated and educated about his condition and prognosis and also what his role would be in the recovery process. Physiotherapy protocols have brought about a good amount of improvement in the patient's status. The patient was relieved from pain to a great extent. Also, the patient was able to ambulate on his own, which has ultimately helped improve the patient's quality of life.
